# Direct evidence for the spin cycloid in strained nanoscale bismuth ferrite thin films

**DOI:** 10.1038/ncomms12664

**Published:** 2016-09-02

**Authors:** Joel Bertinshaw, Ronald Maran, Sara J. Callori, Vidya Ramesh, Jeffery Cheung, Sergey A. Danilkin, Wai Tung Lee, Songbai Hu, Jan Seidel, Nagarajan Valanoor, Clemens Ulrich

**Affiliations:** 1 School of Physics, The University of New South Wales, Sydney, New South Wales 2052, Australia; 2 The Bragg Institute, Australian Nuclear Science and Technology Organisation, Lucas Heights, New South Wales 2234, Australia; 3 School of Materials Science and Engineering, The University of New South Wales, Sydney, New South Wales 2052, Australia

## Abstract

Magnonic devices that utilize electric control of spin waves mediated by complex spin textures are an emerging direction in spintronics research. Room-temperature multiferroic materials, such as bismuth ferrite (BiFeO_3_), would be ideal candidates for this purpose. To realize magnonic devices, a robust long-range spin cycloid with well-known direction is desired, since it is a prerequisite for the magnetoelectric coupling. Despite extensive investigation, the stabilization of a large-scale uniform spin cycloid in nanoscale (100 nm) thin BiFeO_3_ films has not been accomplished. Here, we demonstrate cycloidal spin order in 100 nm BiFeO_3_ thin films through the careful choice of crystallographic orientation, and control of the electrostatic and strain boundary conditions. Neutron diffraction, in conjunction with X-ray diffraction, reveals an incommensurate spin cycloid with a unique [11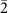] propagation direction. While this direction is different from bulk BiFeO_3_, the cycloid length and Néel temperature remain equivalent to bulk at room temperature.

## Abstract (web summary)

Whilst possessing potential for electrically-controlled magnonic applications, the spin cycloid of bulk BiFeO_3_ is lost in technologically-relevant thin films. Here, the authors demonstrate the stabilization of a long-range spin cycloid in BiFeO_3_ thin films through careful choice of electrostatic and strain conditions during thin film growth.

## Graphical abstract

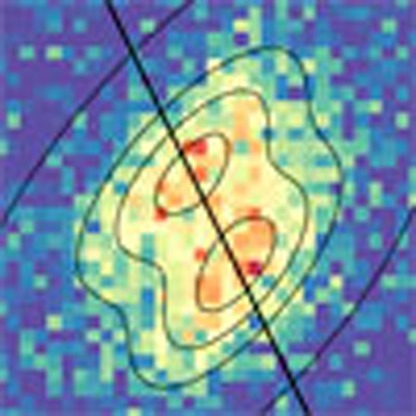

The presence of simultaneous spin and charge order in the eminent room temperature multiferroic BiFeO_3_ (BFO) has spawned a significant research effort to utilize magnetoelectric coupling in this material for new devices designed around thin-film heterostructures^1^,^2^,^3^. BiFeO_3_ is distinctive as a multiferroic as it constitutes the rare case where spontaneous antiferromagnetic (*T*_N_=643 K) and ferroelectric (*T*_C_∼1,123 K) order coexist well above room temperature^4^,^5^. As a Type-I multiferroic, direct magnetoelectric coupling between ferroelectricity and magnetic order in BFO is exceedingly weak^6^. As a result, the primary focus thus far has been aimed at exploiting indirect quasi-static magnetoelectric coupling that arises in composite and layered systems involving BFO^7^,^8^. However, an exciting new possibility opened when linear coupling between ferroelectric order and dynamic spin excitations was demonstrated by Raman light scattering experiments through a systematic electric field control of magnon mode frequencies^9^. More recently a direct linear magnetoelectric coupling effect, driven by the Dzyaloshinsky–Moriya interaction mediated by Fe^3+^ spins forming an incommensurate cycloid, has emerged as an experimental approach to control associated spin waves in BFO via applied electric fields^10^. Electric field control of the cycloidal spin order and associated properties using nanoscale thin films would open new pathways for magnetoelectric memory and spin-wave-based logic gates^11^,^12^,^13^, ensuring practicable voltage ranges and device integrability. This is because manipulation of the cycloid allows control over the magnetoelectric coupling, as the cycloid structure causes a cancellation of the linear magnetoelectric effect that can be revived by suppression of the cycloid^14^. However, the stabilization of a large-scale uniform spin cycloid in nanoscale (∼100 nm) thin BFO films has not been accomplished. Although micron-thick films have already demonstrated promise^15^, the downscaling to nano-dimensions is imperative if one has to realize practically viable magnonic devices. The nanoscale thickness allows several decades faster switching and drive power that is only a small fraction of the thick films, showing potential for compatibility with low-voltage complementary metal-oxide semiconductor circuits.

The long periodicity of the spin cycloid is one of the distinguishing features of the magnetic order in BFO. In bulk BFO the incommensurate spin cycloid structure results from the Fe^3+^ electron spins aligning in a canted G-type antiferromagnetic arrangement. The cycloid is shown to have an extended propagation length of 62–64 nm (refs 16, 17, 18). For the implementation of nanoscale thin BFO films in magnetoelectric or spintronic devices it is desirable that a large-scale spin cycloid is retained. However, the exact electrical and mechanical boundary conditions that stabilize a long-range spin cycloid in nanoscale BFO thin films are yet to be identified. One of the key factors that govern the thin-film BFO behaviour is epitaxial strain through substrate-induced constraint^2^. While the effect of epitaxial constraints on ferroelastic domain formation and polarization switching mechanisms in thin-film BFO is well studied^19^,^20^,^21^,^22^, there has been some debate over the bearing of the epitaxial constraint on the magnetic structure^2^,^19^,^23^,^24^,^25^,^26^,^27^,^28^. Early work proposed that this cycloid should collapse in thin films of <∼300 nm grown on (001) and (111)-oriented STO^23^,^24^,^28^ due to the presence of the large epitaxial strain. Indeed, the cycloid has been shown to exist in much thicker (110)-oriented 1,000 nm films^15^,^27^, and mixed collinear and incommensurate magnetic phases have been indirectly detected in a slightly strained 70 nm thick system grown on (001)-oriented DyScO_3_ (ref. 28).

Essentially, the Fe^3+^ spins in BFO couple strongly to the ferroelectric rhombohedral structural distortion, resulting in an easy magnetic plane that lies perpendicular to the electric polarization vector, establishing an indirect magnetoelectric coupling pathway between ferroelastic and antiferromagnetic domains. In thin films, the easy plane has been shown to reduce to an easy axis either in the [1
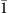
0] or in the [11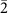] directions^2^,^19^,^25^,^27^,^28^. Thus a key question arises: is it possible to control the presence of the magnetic cycloid in a carefully engineered epitaxial system?

Here we report the retention of the spin cycloid in 100 nm thin films of BFO, grown on (110)-oriented SrTiO_3_ (STO) substrates with a SrRuO_3_ (SRO) bottom electrode. This lifts the previously considered geometrical constraint that the spin cycloid collapses in thin films thinner than ∼300 nm grown on STO substrate^23^,^24^,^28^. Recently we were able to show that careful neutron diffraction measurements with state-of-the-art sensitivity allow for the study of complex magnetic structures even in very thin magnetic films^29^. Exploiting this capability we demonstrate that substrate orientation and electrostatic boundary conditions imposed by the SRO layer can be used to engineer the coupled ferroelastic and antiferromagnetic domain state with an uniform cycloid state in thin-film BFO. In conjunction with X-ray diffraction reciprocal space mapping (RSM), we reveal that the spin cycloid in 100 nm thin nanoscale BFO films propagates along a single unique [11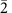] direction with a cycloidal length of *λ*_0_=64.7 nm, matching the value found in bulk BFO. These observations thus reveal that a single-phase spin cycloidal structure can be retained in thin-film BiFeO_3_ grown on STO, opening new avenues of study into the interaction of incommensurate magnetic structure upon interfacial coupling in spintronic heterostructures and controllable magnetoelectric coupling in thin magnonic devices. The reported discovery of a large-scale uniform cycloid in thin-film BFO through careful control of boundary conditions opens new avenues for fundamental research and technical applications that exploit the spin cycloid in spintronic^7^,^8^ or magnonic^9^,^13^ devices. Given the multiferroic nature of BFO, it potentially allows the electric-field manipulation of the spin cycloid in nanoscale BFO films for the first time, and makes it possible to develop future integrated magnonic devices such as spin-wave filters or microwave frequency shifters that can be dynamically manipulated with an applied electric field.

## Results

### Growth and characterization of thin-film BFO

Thin BFO samples with thicknesses of 50, 100 and 150 nm were grown by pulsed laser deposition (PLD) on bare (110)-oriented STO substrate with and without the inclusion of a 20 nm intermediate layer of conductive SRO in order to investigate the combined effects of film thickness and electrostatic growth conditions. X-ray reflectometry (XRR) measurements were performed to provide initial information about the absolute thicknesses of the layers and the roughness of the thin-film interfaces (Supplementary Fig. 1). Reflectivity modelling revealed a root-mean-squared interface roughness of less than 1 nm at the buried interfaces between the compounds, indicative of high-quality film growth. X-ray diffraction polar maps gave clear single crystalline reflections arising from the film, indicating epitaxial growth (Supplementary Fig. 1). The lattice mismatch between the substrate (STO, *a*=3.905 Å (ref. 30)) and film materials (BFO, *a*_pc_=3.968 Å (ref. 31) and SRO, *a*_pc_=3.923 Å (ref. 32), where *pc* denotes pseudo-cubic) results in compressive in-plane strain and an expansion of the out-of-plane lattice parameter. Variation in the strain of the BFO layer with and without the SRO buffer layer was identified using X-ray diffraction RSM (Supplementary Figs 2 and 3), with a consistent lattice *c*/*a* ratio of ∼1.021 at *d*=100 nm with a SRO intermediate layer and ∼1.014 at *d*=100 nm without the SRO layer. In comparison, (001)-oriented films of equivalent thickness exhibit a *c*/*a* ratio of ∼1.04 (ref. 33), indicating that the epitaxial constraints of (110)-orientated films result in an altered relaxation process^34^.

### Spin cycloid in a 100 nm thick bismuth ferrite film

**Figure 1:**
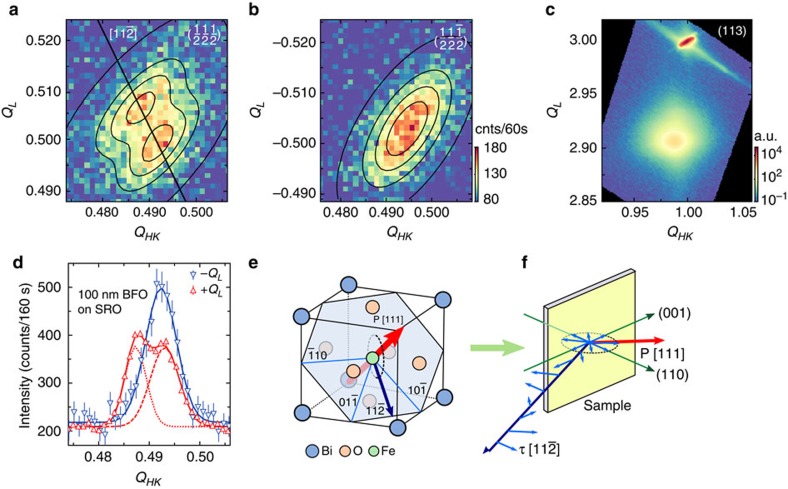
Neutron diffraction data demonstrating the spin cycloid in a 100 nm BiFeO_3_ film. (**a**,**b**) Neutron diffraction reciprocal space maps of a 100 nm BFO film grown on (110)-oriented STO with a 20 nm conductive SRO intermediate layer in the 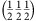 and reversed 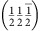 sample orientation. In (**a**) satellite peaks emerge, while in (**b**) a single peak is present, indicative of an incommensurate spin structure. The black line in (**a**) was determined from the peak maxima and indicates the propagation direction of the spin cycloid (**c**) X-ray RSM of the (113) reflection demonstrates single-domain growth of the sample. (**d**) Neutron diffraction diagonal slice through the AFM reflections provides a comparison between the two orientations. The overall measured intensity of the signal is retained in both orientations, that is, the integrated intensity of the satellite peaks around 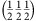 is equal to the 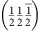 intensity. The error bars correspond to 1 s.d. of the count rate. (**e**,**f**) The neutron diffraction data reveals a unique [11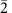] cycloid propagation direction in contrast to [1
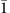
0] as observed in bulk BFO, but with an almost identical spiral length of *λ*_0_=64.7(10) nm as compared to bulk BFO. The cycloidal propagation direction is perpendicular to the direction of the electric polarization P[111] and the in-plane [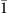
10] direction, resulting in the [11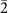] spin cycloid propagation direction which is slightly canted out of the film plane. The light green arrow denotes the direction of the incoming neutron beam.

To determine the precise magnetic structure within the BFO thin films, neutron diffraction RSMs were measured around the G-type antiferromagnetic 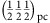 and 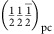 reflections. Samples were aligned with respect to the lattice parameters of the STO substrate, such that the epitaxially strained reflections of BFO appear slightly displaced from ideal positions. Data were initially collected at *T*=300 K, that is, above the ferromagnetic phase transition of SRO at ∼160 K but within the multiferroic phase of BFO. In Fig 1a,b neutron RSM maps of two orientations for the 20 nm SRO/100 nm BFO sample are shown as colour maps with respect to the *Q*_HK_ and *Q*_L_ lattice directions. Incommensurate splitting was identified in the 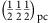 orientation in Fig. 1a, whereas only a single magnetic Bragg peak appears around 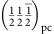 in Fig. 1b. In the diagonal slice of the peak structure for both orientations (Fig. 1d), two separate peaks were found for the 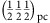 orientation with an equal distribution of the integrated intensities, while the intensity of the single magnetic Bragg peak observed around 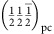 matches the intensity of both magnetic satellite peaks in the other orientation.

In bulk BFO the cycloidal spin structure propagates in one of three directions in the magnetic easy plane, which manifests as an incommensurable splitting of the magnetic Bragg reflection in the [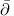
*∂*0], [0*∂*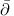], [*∂*0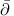] directions for the [111] electric polarization, with *∂*=0.0045 reciprocal lattice units (rlu) (indicated as light blue lines in Fig. 1e, see also refs 18, 35). In the case of an epitaxial thin-film system, in-plane strain lifts the degeneracy of the magnetic structure, and the easy magnetic plane collapses into an easy axis^2^,^19^,^23^,^27^. A signature of the spin cycloid is thus an incommensurate splitting in only one crystallographic orientation, corresponding to the easy magnetic axis in which the cycloid propagates. Such a behaviour is clearly identifiable in Fig. 1, where the incommensurate splitting arises only around the 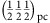 reflection. On the other side, a peak splitting of the antiferromagnetic reflection may also arise due to indirect magnetoelectric coupling between multiple ferroelastic domain types. To ensure that the 100 nm SRO/BFO film is an untwinned single domain, a X-ray RSM around the (113) reflection was conducted (Fig. 1c). The presence of a single reflection corresponding to the BFO film demonstrates the single structural domain nature of the entire film. This result is underlined by piezoresponse force microscopy measurements (Supplementary Fig. 4), which indicates an as-grown single ferroelectric domain state.

The double-peak structure around the 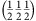 orientation shown in Fig. 1a was fitted with two-dimensional (2D) Gaussian functions. The results are depicted in Fig. 1a as contour lines. Note that the elliptical nature of the Bragg reflections is caused by the finite instrumental resolution, which is not isotropic for different directions in **Q**-space. Using the centre of the mass of each peak, the propagation vector and magnitude of the incommensurate splitting corresponding to the magnetic easy axis and concomitant spin cycloid propagation direction was determined. The observed splitting size of ∂=0.00425 rlu corresponds to a spin cycloid length of *λ*_0_=64.7(10) nm, which matches the value observed in bulk BFO. Additional polarized neutron diffraction experiments using low-pressure nuclear polarized ^3^He gas cells have indicated that selectively one of the two peaks, shown in Fig. 1d for the 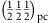 reflection is present when using the +− and −+ polarized neutron configurations for the incident and scattered beam with the direction of the neutron spins parallel to the momentum transfer of this Bragg reflection (Supplementary Fig. 5). This is consistent with the observation of Ratcliff II *et al.*^15^ and is a consequence of the chirality of the spin structure. The direction of the scattering vector, shown as a solid line in Fig. 1a, lies along the [11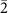] direction (Fig 1e,f) and is distinctively different to the propagation direction [1
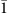
0] of the spin cycloid in bulk BFO. The same [11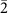] spin cycloid propagation direction was observed in significantly thicker BFO films (*t*=1,000 nm) grown on (110)-oriented STO, but with an altered cycloidal length of 59 nm (ref. 27). The effect of compressive in-plane epitaxial strain imposed by the STO substrate affects the degeneracy of the magnetic easy plane. Based on prior first-principles calculations of the magnetocrystalline anisotropy of BFO^19^, it was deduced that for thin-film BFO under compressive strain on (001)-orientated STO the easy antiferromagnetic axis is perpendicular to the rhombohedral axis and simultaneously parallel to the out-of-plane (001) direction, which results in the [1
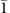
0] propagation of the spin cycloid for an electric polarization along the [111] direction. In contrast, Holcomb *et al.*^25^ suggest that for compressively strained BFO films, the magnetic easy axis shows a preference for the direction with the largest out-of-plane component, while remaining perpendicular to the rhombohedral axis, which lies along the [11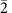] direction in (001)-oriented films. In the present study, for the modified epitaxial constraints present in an (110)-oriented film, the [11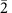] direction corresponds to an easy axis that lies perpendicular to the rhombohedral axis and parallel to the out-of-plane (110), confirming the theory presented by Zhao *et al.* in ref. 19.

The deposition of epitaxial thin-film BiFeO_3_ can be controlled through the careful choice of growth conditions including substrate type, orientation and layer composition. The non-centrosymmetric rhombohedral perovskite structure of bulk BFO (space group *R*3*c*) results in eight domain variants, which correspond to positive and negative polarizations along four structurally distinct ferroelastic distortion types^36^. In thin-film form, BFO grown on (001)-oriented STO substrate typically retains the four structurally distinct twin domain patterns and (111)-oriented systems exhibit bulk-like single-domain formation^37^. The use of (110)-oriented STO enforces two out of the four possible structurally distinct ferroelectric domain types^21^. The inclusion of a conducting SRO buffer layer improves the quality of thin-film BFO and leads to a ferroelastic mono-domain growth, as found in the 100 nm SRO/BFO sample discussed in Fig. 1. In addition to reducing the number of substrate terraces that favour the formation of certain domain walls^38^,^39^, the metallic behaviour of SRO provides the potential for an electrostatically favourable environment for single-domain growth of BFO^38^,^39^. As a consequence, the spin cycloid does not collapse in the 100 nm thin BFO film grown on SRO/STO.

### Magnetic Structure of a BFO film without intermediate layer

**Figure 2:**
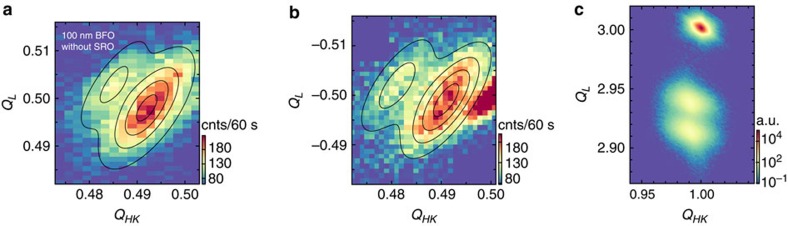
Neutron diffraction data of a 100 nm BiFeO_3_ film grown without a conductive intermediate layer. X-ray and neutron reciprocal space map of the (110)-oriented 100 nm BiFeO_3_ sample without an intermediate SRO layer. (**a**,**b**) Show the neutron diffraction data taken around 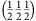 and 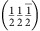, respectively. (**c**) Presents the corresponding X-ray RSM around the (113) reflection, demonstrating the existence of two structural domains. The different displacements of the Bragg reflections with respect to the Q_HK_ and *Q*_L_ values arises from the fact that neutron diffraction measurements were performed in a geometry out-of-plane, whereas X-ray RSM maps were collected in-plane.

To further investigate this observation, (110)-oriented samples with 100 and 150 nm BFO film thickness were fabricated without the intermediate SRO electrode on STO substrates. Figure 2 shows neutron and X-ray RSMs of this 100 nm BFO film. The neutron diffraction RSMs show a double-peak structure for both 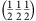 and 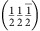 orientations. The same double-peak structure is reflected in the corresponding X-ray data, which was taken around the (113) structural Bragg peak (Fig. 2c). In addition, piezoresponse force microscopy measurements clearly show a two domain pattern (Supplementary Fig. 4). The double-peak structure observed in the neutron diffraction data exhibits distinct differences to the previous BFO sample with the SRO electrode: the twin peak structure is clearly visible in both orientations in the neutron and X-ray data sets, the splitting of the peak forms directly along the [111] direction, and the intensity of both peaks is not equal. Altogether, these differences indicate that the 100 nm sample grown without metallic SRO exhibits two distinct ferroelastic twins. Two domain variants were also identified with a similar intensity mismatch in the 150 nm BFO sample (Supplementary Fig. 6). It should be noted that the additional peak at 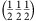 in Fig. 2b) arises from second order contamination as a consequence of a reduced amount of pyrolytic graphite in the neutron beam path.

**Figure 3:**
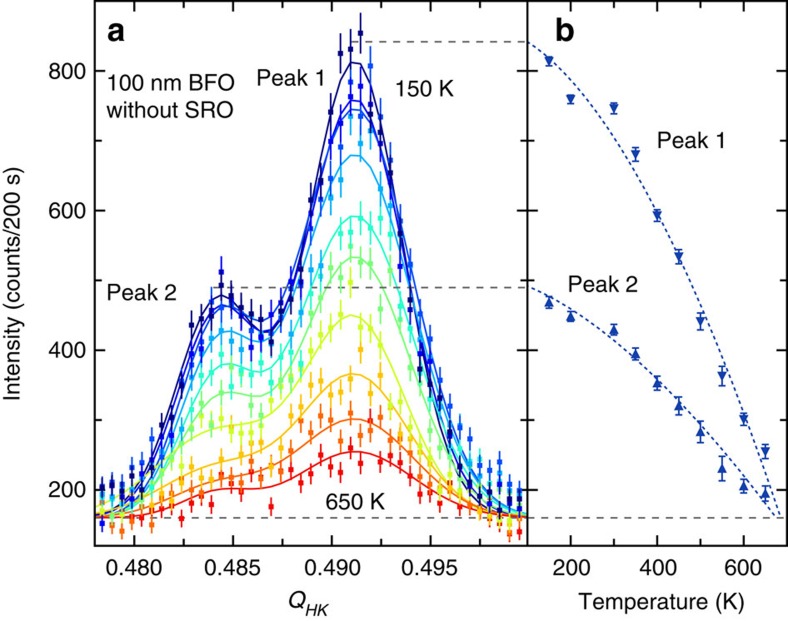
Temperature dependence of the magnetic signal. (**a**) Temperature dependency up to 650 K in (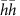
*l*) slices of the 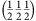 peak structure. (**b**) The extrapolation using a power law behaviour of the fitted peak intensity of the two peaks reveals *T*_C_≈650 K, which corresponds well to the transition temperature of bulk BFO. The error bars in (**a**) correspond to 1 s.d. of the count rate while in (**b**) they correspond to 1 s.d. of a Gaussian fit to the entire diffraction peak.

To demonstrate the magnetic origin of the observed double-peak structure in the neutron RSM data, the temperature dependence of the signal of the 100 nm BFO film without the SRO buffer layer was taken from 200 K up to 650 K. Scans were performed in the (*hk*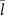) direction with 50 K steps, and are shown in Fig. 3a). The resulting temperature dependency of the peak intensities are shown in Fig. 3b). Both peaks exhibit the same gradual trend and have almost entirely disappeared at 650(10) K. This is in good agreement with the magnetic phase transition temperature 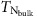
=643 K of bulk BFO (ref. 4) and indicates that the epitaxial strain, which could result in antiferrodistortive rotations of the FeO_6_ oxygen octahedra in the distorted rhombohedral perovskite structure, does not have a significant effect on the primary exchange interaction between Fe^3+^ ions in BiFeO_3_ down to a thickness of 100 nm. Since the observed separation of the peaks is similar to the incommensurate splitting for of the cycloidal spin structure (Fig. 1) the additional complexity in the neutron diffraction signal makes it difficult to ascertain any conclusive information about the presence of the incommensurate spin-cycloid, or the potential for a coexistence of a spin spiral and pure G-type antiferromagnetic ground state, as identified in 70 nm BFO grown on (001)-oriented DyScO_3_ by Sando *et al.*^28^.

### Absence of the spin cycloid in a 50 nm thin BFO film

**Figure 4:**
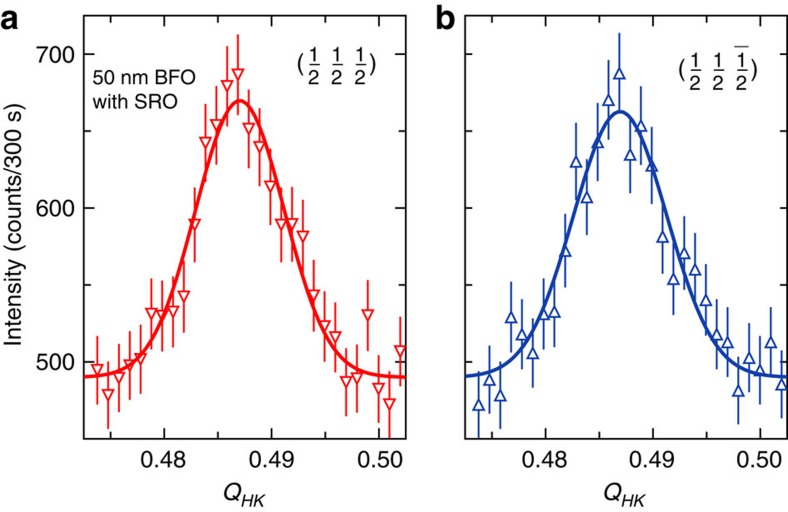
Absence of the spin cycloid in a 50 nm BiFeO_3_ film. (**a**,**b**) Neutron diffraction conducted on a (110)-oriented 20 nm SRO/50 nm BFO sample in the 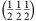 and 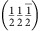 orientations shows a single-peak structure, indicating that the spin cycloid is suppressed. The error bars correspond to 1 s.d. of the count rate.

In contrast to the cycloidal spin arrangement in the 100 nm BFO film grown with an SRO intermediate layer shown in Fig. 1, the spin cycloid collapses to a collinear G-type antiferromagnetic structure in a 50 nm BFO thin film with an intermediate 20 nm conducting SRO layer. In Fig. 4 it is clearly evident that in all accessible 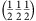 orientations no incommensurate peak formation arises. A X-ray diffraction RSM of the (113) peak reveals that like the 100 nm sample SRO/BFO sample only a single structural domain variant has formed during film growth. Large epitaxial strain leads to the collapse of the spin cycloid in BFO^23^,^24^. A recent systematic investigation using Mössbauer spectroscopy and Raman light scattering indicated boundaries of the epitaxial in-plane strain of <−1.7 and >+0.5% for the transition of the cycloidal spin arrangement to a collinear G-type antiferromagnetic structure^28^. For BFO films grown on (001) oriented STO substrate with a strain state of −1.7% the epitaxial strain relaxes for a film thickness of larger than 100 nm^33^. To determine the in-plane and out-of plane strain within the BFO thin-film samples, X-ray RSM maps were performed around the (220) reflection (Supplementary Figs 2 and 3). The out-of-plane strain did relax from about −1.5% for the 50 and 100 nm BFO films grown on an SRO intermediate layer to about −1.2% for the 100 and 150 nm BFO film samples grown without the SRO intermediate layer. These values are smaller than the strain reported in films grown on (001) oriented STO substrate^33^, as expected since alternate relaxation processes arise in (110) films due to the modified epitaxial constraints^34^.

## Discussion

Our investigation demonstrates that the electrostatic conditions and crystallographic orientation play a vital role in determining the magnetic ground state in nanoscale BFO thin films. Particularly here, the 100 nm BFO film grown with an SRO intermediate layer exhibits a higher level of strain than the 100 nm BFO film without SRO. Critically, it retains a large-scale cycloidal spin arrangement within a ferroelastic monodomain state. Without the SRO layer, the 100 and 150 nm BFO films accommodate for the in-plane stress by relaxing into a two-domain state and exhibit a smaller out-of-plane lattice parameter. However, the spin cycloid is only clearly present in the 100 nm sample with SRO, despite the increased level of epitaxial strain present. This challenges the current paradigm of a strain induced collapse of the spin spiral in thin BFO films grown on STO^23^. When this strain is further increased, as found in the 50 nm film, the cycloid collapses into collinear G-type antiferromagnetic order, as indicated by the single diffraction peak in Fig. 4. Therefore, electrostatic conditions not only play a defining role in determining the ferroelectric domain structure^40^ but also the magnetic ground state in bismuth ferrite.

This demonstrates that oxide heteroepitaxy of bismuth ferrite can be carefully tuned to achieve elusive and exotic ferroic ground states. We have revealed that the epitaxial constraints of a (110)-oriented substrate, in conjunction with the presence of an intermediate conductive SRO buffer layer, can stabilize a uniform long-range spin cycloid with a unique propagation direction across the entire film. The presence of incommensurate magnetic satellite peaks, mapped via neutron diffraction, without a corresponding structural splitting, as identified with X-ray diffraction, confirm the spin cycloid and indicate a cycloid propagation length *λ*_0_=64.7 nm, which is consistent with the bulk value. Thus, under optimal epitaxial conditions the magnetic ground state is robust even for a strained 100 nm BFO layer. In contrast, (001)-oriented films grown on STO exhibit the well-known coupled ferroelastic and antiferromagnetic domain structure that leads to a breakdown of the spin cycloid (for example, see ref. 23). This underlines the importance of the correct mechanical and electrical boundary conditions required to achieve emergent spin properties in mutiferroic thin-film systems.

In summary we demonstrate that the stabilization of a large-scale uniform spin cycloid is possible in nanoscale (100 nm) thin BiFeO_3_ films by exploiting crystallographic, epitaxial and electrical boundary conditions. Although this is only the first step, it is the most crucial in the long and complex task of making a fully integrated magnonic device. Given the unique crystallographic direction of the spin wave propagation, it now becomes possible to couple the chirality of the spin cycloid to electric field (that is an applied electric field would change the polarization direction which in turn would have ramifications on the crystallographic orientation) in nanoscale BiFeO_3_ films, which opens up intriguing possibilities for magnetoelectrically coupled spin-wave devices.

## Methods

### Sample preparation and characterization

Thin-film samples of varying BFO film thickness between 50 and 150 nm were grown using pulsed laser deposition on (110)-oriented STO substrates with ∼8 × 8 mm^2^ in size with and without a 20 nm SRO buffer layer. A Neocera PLD system with a 248 nm wavelength KrF excimer Laser was used to deposit atomic precise layer by layer^41^. An oxygen partial pressure of 10 mTorr and substrate temperatures of 850 and 800 °C were held for the deposition of the BFO and SRO layers, respectively. The deposited systems were cooled in a partial oxygen pressure of 200 mTorr at a rate of 20 K min^−1^. X-ray reflectivity (XRR) was conducted using a X'Pert Pro Instrument by Phillips with Cu-K*α* radiation. X-ray RSM were performed using a Bruker D8 TXS, set up using a Lynxeye 1D detector, 6 kW Cu-K*α* radiation source, and four bounce Ge (220) monochromators.

To verify epitaxial growth of the BFO thin-film samples, X-ray reflection (XRR) and X-ray polar maps were measured (Supplementary Fig. 1). The XRR measurements gave clear Kiessig fringes and were analysed by fitting a layer structure including the roughnesses at the interface to the substrate and to air to the experimental data using the MOTOFIT software suite^42^. This revealed root-mean-squared interface roughnesses of <1 nm for the buried interfaces within the layer and at the film-to-air interface of all investigated samples. The X-ray polar maps around the (113) reflection (Supplementary Fig. 1) were taken on a Bruker D8 TXS diffractometer. No additional Bragg peaks were detected, thus ensuring the epitaxial nature of all films.

### Neutron diffraction

To investigate the magnetic structure of the BFO layer, neutron diffraction was performed on the triple-axis spectrometer Taipan, located at the Opal research reactor at ANSTO in Sydney, Australia. Taipan provides the strong signal-to-noise ratio, necessary for measuring nanometre thickness thin films with neutron diffraction^29^. The instrument was set up for standard elastic diffraction with a vertically focussed highly ordered pyrolytic-graphite monochromator, with a fixed wavelength of *λ*=2.346 Å. The pyrolytic-graphite analyser was also set in a vertical focussing configuration, with 40′ collimation to achieve the required resolution. The BFO films were oriented within the [110] × [001] scattering plane in order to access the 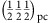 reflections. Second-order diffraction contaminations emanating from the much thicker substrate were removed from the scattered signal with the inclusion of two pyrolytic graphite filters of 60 mm total thickness in the instrument configuration. For polarized neutron diffraction experiments low-pressure ^3^He gas cells, which were polarized using a laser system that pumps discharge-excited helium atoms into a spin-polarized state which subsequently polarizes the ^3^He nuclei through hyperfine interaction, were placed into the incident and scattered neutron beam. A magnetic field parallel to the momentum transfer **Q** of the corresponding Bragg reflection was applied at the sample position using a vector magnet. All experiments were performed at room temperature unless otherwise indicated.

### Data availability

The data that support the findings of this study are available from the corresponding author upon request.

## Author contribution

J.B., S.J.C., S.A.D., W.T.L. and C.U. participated in the neutron diffraction experiment and performed the data analysis. R.M., V.R., J.C., S.H., J.S. and N.V. prepared and characterized the samples. J.S., N.V. and C.U. conceived and supervised the project. J.B., J.S., N.V. and C.U. wrote the manuscript. All authors contributed to the manuscript and the interpretation of the data.

## Additional information

**How to cite this article:** Bertinshaw, J. *et al.* Direct evidence for the spin cycloid in strained nanoscale bismuth ferrite thin films. *Nat. Commun.* 7:12664 doi: 10.1038/ncomms12664 (2016).

## Supplementary Material

Supplementary Information Supplementary Figures 1-6 and Supplementary References.
