# Supplementary material for: Direct evidence for the spin cycloid in strained nanoscale bismuth ferrite thin films
Source: Nat Commun. 2016 Sep 2;7:12664. doi: 10.1038/ncomms12664 (PMC5025793; doi:10.1038/ncomms12664)
Supplement: Supplementary Information — Supplementary Figures 1-6 and Supplementary References. [file ncomms12664-s1.pdf]

Supplementary Figures

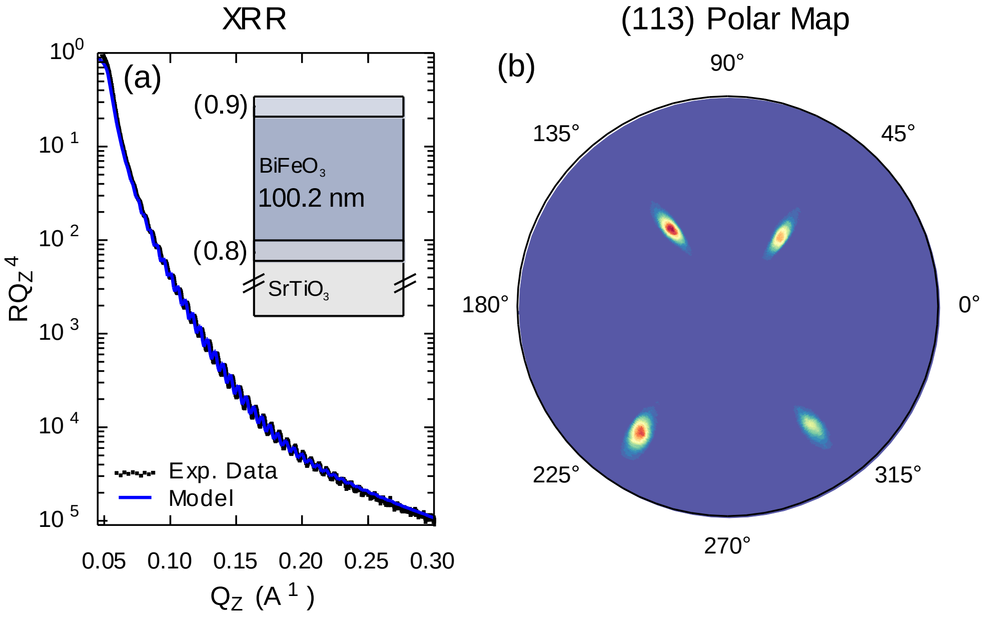

Supplementary Figure 1 | X-ray data of the 100 nm $BiFeO_3$ film without conductive $SrRuO_3$ intermediate layer. (a) X-ray reflectivity and (b) (113) polar map of the 100 nm BFO film without SRO indicates pseudomorphic epitaxial growth and a small interface roughness of 0.8 nm at the STO substrate and 0.9 nm at the film to air interface.

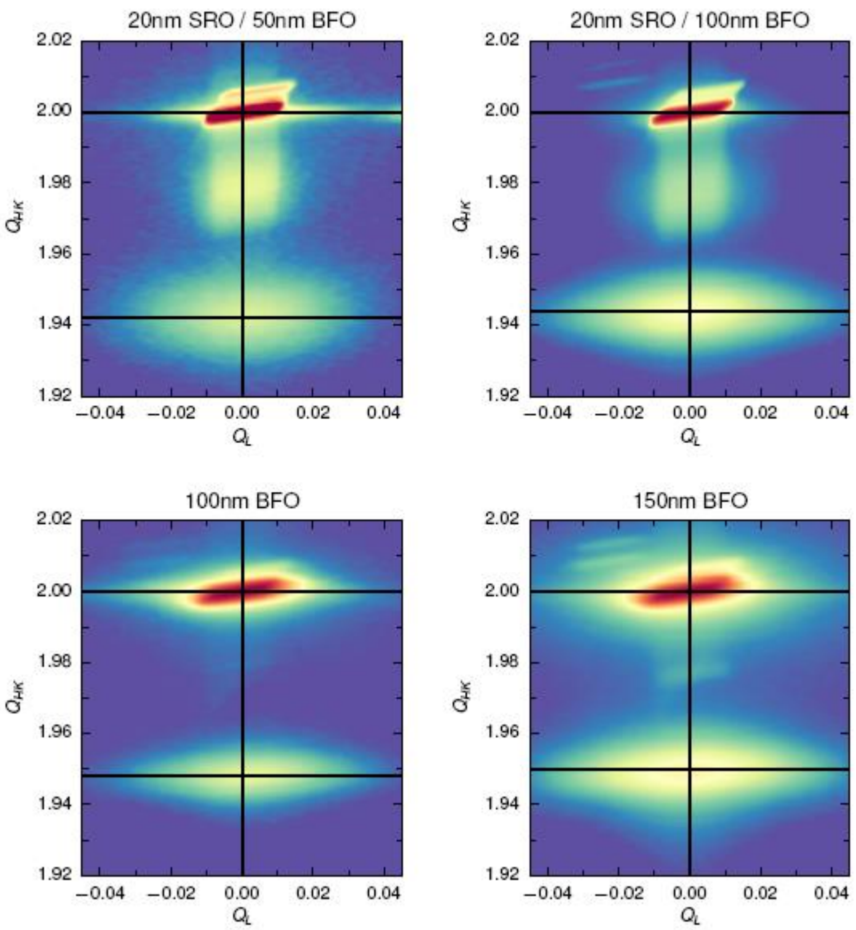

Supplementary Figure 2 | X-ray reciprocal space maps around the (220) reflection. X-ray RSMs around the (220) pseudocubic structural reflection of the (110)-oriented thin film samples presented in the main text and the Supplementary Figures. The horizontal lines indicate the location of the (220) Bragg reflection of the STO substrate and the BFO film at around $\mathbf{Q_{HK}}$ = 1.94-1.95. Due to epitaxial growth the crystal lattice of the BFO and SRO layers with initial lattice parameters of a_{pc} = 3.968 Å [1] and a_{pc} = 3.923 Å [2], respectively, are clamped in-plane to the substrate lattice parameter (STO, a = 3.905 Å [3]), resulting in an in-plane compressive strain and an expansion of the out-of-plane lattice parameter. The obtained out-of-plane lattice parameters of the BFO films grown on (110) oriented STO are shown in the Supplementary Figure 3.

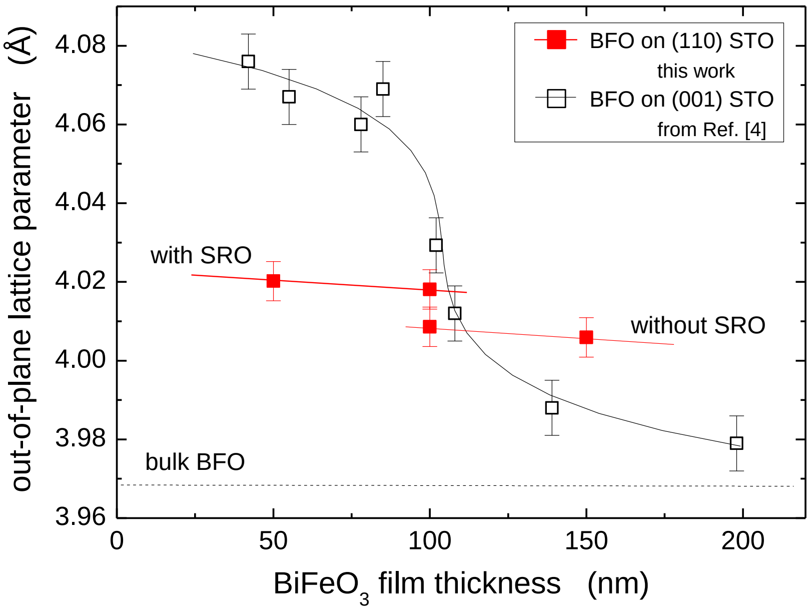

Supplementary Figure 3 | Out-of-plane strain relaxation in $BiFeO_3$ thin films. The out-of-plane pseudocubic lattice parameter of the BFO samples of this study grown on (110)-oriented STO substrate are plotted as a function of film thickness as red squares. For comparison, previously reported data by Kim et al. [4] of BFO thin films on (001)-oriented STO substrate are shown as open black symbols. The lines serve as guides to the eye. The difference between the out-of-plane lattice parameters for the films grown on (110) and (001) oriented substrates results from the modified epitaxial constraints between the two types of films. While BFO films grown on (001)-oriented STO can compensate the epitaxial strain by expanding in (001) direction, films grown on (110) can expand along both the (110) and (100) directions, enabling an additional route to relaxation, while simultaneously enforcing a two-variant domain growth [5].

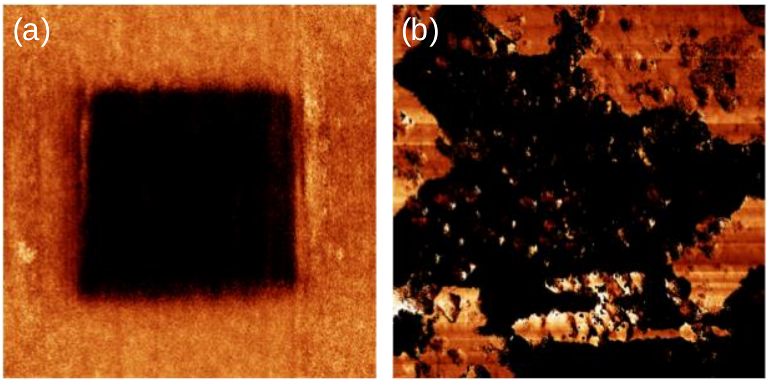

Supplementary Figure 4 | Piezoresponse force microscopy of the domain structure of $BiFeO_3$ thin films. (a) Out-of-plane piezoresponse force microscopy image of the monodomain structure of the 100 mn BFO film grown with a conductive intermediate SRO layer on (110) oriented STO. The ferroelectric state could be switched electrically using an atomic force microscope tip, resulting in the darkened square region in the centre. (b) In-plane piezoresponse force microscopy image of the 100nm BFO film without SRO in as grown state is showing two in-plane domain variants.

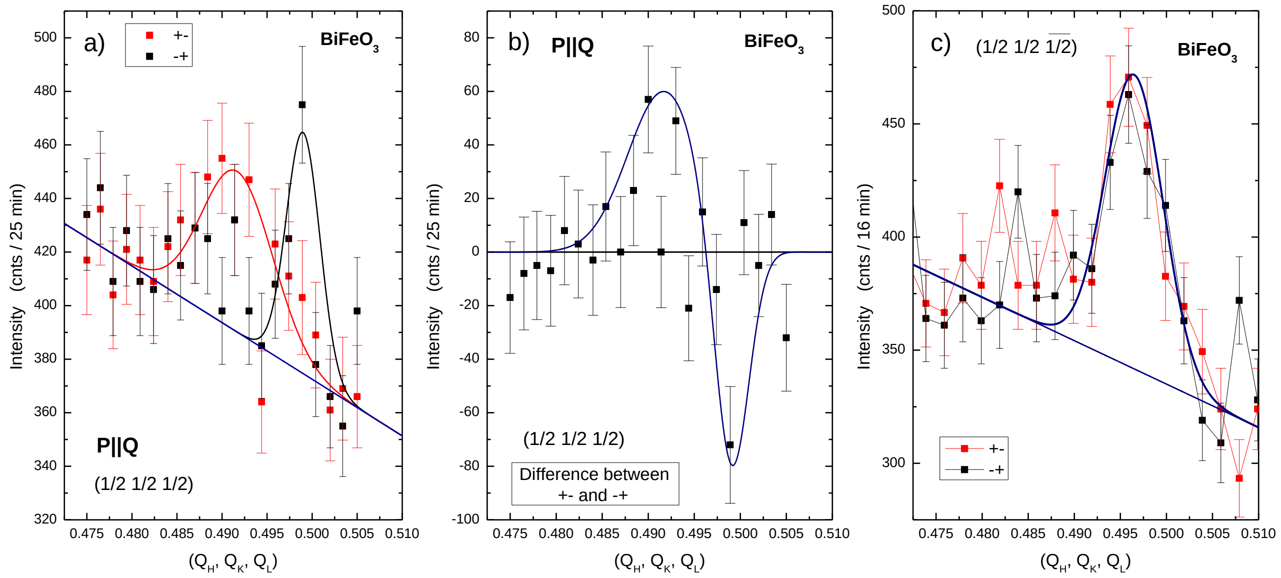

Supplementary Figure 5 | Polarized neutron diffraction data verifying the spin cycloid in thin film $BiFeO_3$. (a) Polarized neutron experiment of the 100 nm BFO film grown on (110)-oriented STO substrate with a 20 nm conductive SRO intermediate layer around the (½ ½ ½) sample orientation. The + and – notation correspond to the polarization state for the incident and scattered beam, respectively. A magnetic field parallel to the scattering vector **Q** of the corresponding Bragg reflection was applied at the sample position. An asymmetry between both polarization channels +- (red) and -+ (black) is present, demonstrating the existence of a chirality axis along the [11-2] direction. (b) The difference in intensity between both polarization channels +- and -+ highlights the asymmetry between both peaks. (c) polarized neutron data around the (½ ½ -½) region. In this sample orientation only one magnetic Bragg reflection is expected, indicating that the sample is single domain. The intensity of the two incommensurate Bragg peaks around (½ ½ ½) is almost identical for both polarization conditions +- and -+ and their combined intensity matches the intensity of the single magnetic peak at (½ ½ -½). The error bars correspond to one standard deviation of the count rate. It should be noted that neutron scattering is a volume sensitive technique. Therefore, neutron diffraction on nanoscale thin films is already challenging. The use of polarized neutron further reduces the neutron flux by one order of magnitude.

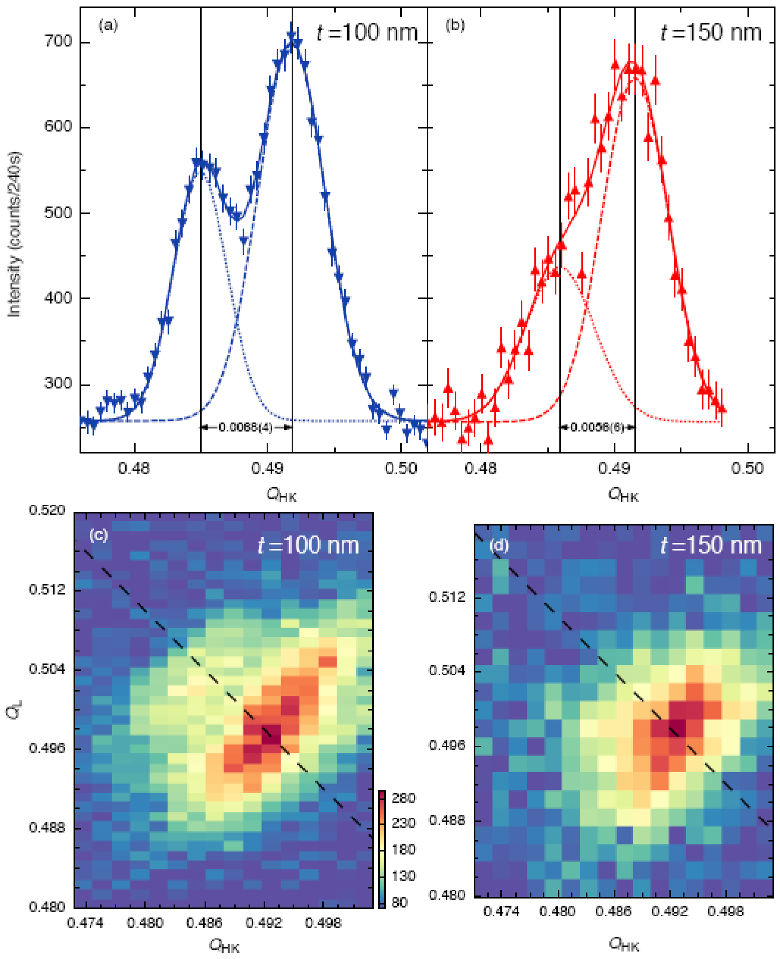

Supplementary Figure 6 | Magnetic structure of $BiFeO_3$ films without metallic intermediate layer. Neutron diffraction data of the (a,c) 100 nm and (b,d) 150 nm thick BFO film grown on (110)-oriented STO without the conductive SRO intermediate layer. (a,b) Cuts through the magnetic Bragg reflection at (½ ½ ½) along the dashed lines in (c,d), reveal a similarly twinned antiferromagnetic domain structure for both samples.

Supplementary References:

1. Moreau, J.-M., Michel, C., Gerson, R. & James. W. J., Ferroelectric $BiFeO_3$ X-ray and neutron diffraction study. *J. Phys. Chem. Solids* **32**, 1315-1320 (1971).
2. Jones, C. W., Battle, P. D., Lightfoot, P. & Harrison. W. T., A., The structure of $SrRuO_3$ by time-of-flight neutron powder diffraction. *Acta Cryst.* **C45**, 365-367 (1989).
3. Lytle. F. W., X-Ray Diffractometry of Low-Temperature Phase Transformations in Strontium Titanate. *J. Appl. Phys.* **35**, 2212-2215 (1964).
4. Kim, D. H., Lee H. N., Biegalski, M. D. & Christen. H. M., Effect of epitaxial strain on ferroelectric polarization in multiferroic $BiFeO_3$ films. *Appl. Phys. Lett.* **92**, 012911 (2008).
5. Li, J., Wang, J., Wuttig, M., Ramesh, R., Wang, N., Ruette, B., Pyatakov, P., Zvezdin, K. & Viehland. D., Dramatically enhanced polarization in (001), (101), and (111) $BiFeO_3$ thin films due to epitiaxial-induced transitions. *Appl. Phys. Lett.* **84**, 5261-5261 (2004).
